# Neuroimaging characterization of multiple sclerosis lesions in pediatric patients: an exploratory radiomics approach

**DOI:** 10.3389/fnins.2024.1294574

**Published:** 2024-02-02

**Authors:** Ricardo Faustino, Cristina Lopes, Afonso Jantarada, Ana Mendonça, Rafael Raposo, Cristina Ferrão, Joana Freitas, Constança Mateus, Ana Pinto, Ellen Almeida, Nuno Gomes, Liliana Marques, Filipe Palavra

**Affiliations:** ^1^Neuroimaging and Biomedicine Research Group, Medical Imaging and Radiotherapy Research Unit, CrossI&D: Lisbon Research Center, Portuguese Red Cross Higher Health School (ESSCVP), Lisbon, Portugal; ^2^Faculty of Science, Institute of Biophysics and Biomedical Engineering, University of Lisbon, Lisbon, Portugal; ^3^Biomedical Research Group, Faculty of Engineering, Faculty of Veterinary Medicine NICiTeS, Lusófona University, Lisbon, Portugal; ^4^Centre for Child Development – Neuropediatrics Unit, Hospital Pediátrico, Centro Hospitalar e Universitário de Coimbra, Coimbra, Portugal; ^5^Laboratory of Pharmacology and Experimental Therapeutics, Faculty of Medicine, Coimbra Institute for Clinical and Biomedical Research, University of Coimbra, Coimbra, Portugal; ^6^Clinical Academic Center of Coimbra, Coimbra, Portugal

**Keywords:** multiple sclerosis, pediatric age, neuroimaging, neuroinflammation, radiomics, magnetic resonance imaging, characterization and classification of lesions

## Abstract

**Introduction:**

Multiple sclerosis (MS), a chronic inflammatory immune-mediated disease of the central nervous system (CNS), is a common condition in young adults, but it can also affect children. The aim of this study was to construct radiomic models of lesions based on magnetic resonance imaging (MRI, T2-weighted-Fluid-Attenuated Inversion Recovery), to understand the correlation between extracted radiomic features, brain and lesion volumetry, demographic, clinical and laboratorial data.

**Methods:**

The neuroimaging data extracted from eleven scans of pediatric MS patients were analyzed. A total of 60 radiomic features based on MR T2-FLAIR images were extracted and used to calculate gray level co-occurrence matrix (GLCM). The principal component analysis and ROC analysis were performed to select the radiomic features, respectively. The realized classification task by the logistic regression models was performed according to these radiomic features.

**Results:**

Ten most relevant features were selected from data extracted. The logistic regression applied to T2-FLAIR radiomic features revealed significant predictor for multiple sclerosis (MS) lesion detection. Only the variable “contrast” was statistically significant, indicating that only this variable played a significant role in the model. This approach enhances the classification of lesions from normal tissue.

**Discussion and conclusion:**

Our exploratory results suggest that the radiomic models based on MR imaging (T2-FLAIR) may have a potential contribution to characterization of brain tissues and classification of lesions in pediatric MS.

## 1 Introduction

Multiple sclerosis (MS) is a chronic neurological disease that mainly affects adults, but which can also manifest itself in children, although it is less common in this age group (Kornbluh and Kahn, 2023). This disease is an immune-mediated condition, being characterized by inflammation and destruction of myelin, the protective layer that surrounds nerve fibers in the central nervous system (CNS) (Teleanu et al., 2023). When MS occurs in children and adolescents (i.e., before 18 years of age), diagnostic challenges are unique, but every expression of the disease in this age group is also unique, as it can affect physical and cognitive development at a crucial time in their lives, the impact on functional prognosis being very important **(Teleanu et al., 2023)**. This condition is thus known as pediatric-onset MS (POMS). It has some distinct characteristics and a different course, compared to adults (one of the most important aspects to mention is the fact that almost all children and adolescents with the diagnosis progress through relapses and remissions) (Alroughani and Boyko, 2018).

In addition to all the importance it has for diagnosis, magnetic resonance imaging (MRI) has allowed the performance of quantitative analysis of white matter and gray matter lesions, being a fundamental imaging modality in clinical research (Harrison et al., 2016). The characteristics of lesions in MS are quite heterogeneous (both in clinical and neuroradiological terms), with different stages of evolution that are related to acute and chronic neuroinflammation processes (active and inactive) (Lucchinetti et al., 2000; Harrison et al., 2016). This heterogeneity makes radiomics the ideal tool to extract and characterize MS lesions, generating tools that could be useful in the eventual automation of differential diagnosis with other types of lesions of the brain parenchyma. Several efforts have been made to apply radiomics in Neurology, namely in the study of tumors, ischemic stroke, thrombosis characterization, identification of high-risk carotid plaques, prediction of malignant middle cerebral artery infarction, intracranial hemorrhage, and intracranial aneurysm (Sotoudeh et al., 2021).

In the field of primary demyelinating disorders, such as MS, where acute or chronic inflammation and reactive astrogliosis may be found, the following diseases have already been studied using radiomics: neuromyelitis optica spectrum disorders (NMOSD), Marburg-type MS, Balo’s concentric sclerosis, as well as acute disseminated encephalomyelitis (ADEM) (Höftberger and Lassmann, 2017). The results obtained were encouraging and this has been seen as a field of clear interest for radiomics research, since it allows the extraction of a large amount of information invisible to human eye, through the definition of regions of interest (ROI), which later allow the development of different models, that can be used as predictors of disease course (Sotoudeh et al., 2021).

In this study, a quantitative and radiomic characterization of POMS lesions was performed, also involving brain tissue around them (transitional brain tissue) and brain tissue without lesions. Volumetric analysis of the brain and lesions were also performed. A radiomics model based on T2-weighted-Fluid-Attenuated Inversion Recovery (T2-FLAIR) images was constructed for the classification of brain tissue in POMS patients scans. Correlations were also analyzed between demographic, clinical, laboratory and radiomics data.

## 2 Materials and methods

### 2.1 Patients

Eleven patients diagnosed with POMS (mean age 17.07 ± 1.6 years; 4 males and 7 females) were included in this study, with an age at diagnosis of 15.6 (± 0.9) years. They were searched and randomly extracted from the database of the Pediatric Demyelinating Diseases consultation in our center. All of them (and their parents) signed an informed consent document to participate in this study, which was previously approved by the local Ethics Committee (Centro Hospitalar e Universitário de Coimbra).

A Neurological examination was performed on all participants and the Expanded Disability Status Scale (Lublin et al., 2014) (EDSS score) was used to assess the degree of disability associated with the disease (mean ± SD = 1.7 ± 0.8). Oligoclonal bands in cerebrospinal fluid (CSF) were detected in 7 patients (63.6%) and were absent in 1 (9.1%) (3 patients were not initially evaluated in our hospital, and information regarding the CSF study was not available). These immunoglobulin G (IgG) bands, detected by isoelectric focusing, indicate intrathecal synthesis of immunoglobulins and may even be useful for differential diagnosis (Deisenhammer et al., 2019; Cabrera, 2022). Demographic, clinical, and laboratorial data of patients included in the study are summarized in Table 1.

**TABLE 1:** Demographic, clinical and laboratorial characteristics.

	*n*	Mean
Age at diagnostic (years)	11	15.6 (± 0.9)
Patient age (years)	11	17.07 (± 1.6)
EDSS score	10	1.7 (± 0.8)
	**M**	**F**
Gender (M/F)	4 (36.4%)	7 (63.6%)
	**Positive**	**Negative**
Oligoclonal bands	7 (63.6%)	1 (9.1%)

EDSS, Expanded Disability Status Scale; Data presented as mean (± standard deviation).

### 2.2 MRI scanning and protocol

In this study, neuroimaging data acquisition was conducted using 1.5 tesla clinical scanners. Brain imaging data were obtained through the utilization of FLAIR sequences on three distinct MR systems: Philips Achieva (TR = 11,000 ms; TE = 140 ms; TI = 2,800 ms; 24 slices with voxel size 0.4 mm × 0.4 mm × 5.9 mm), GE Signa HDxt (TR = 9,502 ms; TE = 123.66 ms; TI = 2,250 ms; 22 slices with voxel size 0.4 mm × 0.4 mm × 5.9 mm), and Siemens Espree (TR = 8,000 ms; TE = 113 ms; TI = 2,500 ms; 24 slices with voxel size 0.4 mm × 0.4 mm × 5.8 mm). These acquisitions were performed to enhance the understanding of neuroanatomical features and to facilitate comprehensive analyses within the scope of this research. The clinical brain MR acquisition protocol performed included at least T1-weighted images (T1-WI), T2-weighted images (T2-WI) and T2-weighted-Fluid-Attenuated Inversion Recovery (T2-FLAIR) sequences. Comparison of pre- and post-gadolinium (Gd) in T1-WI was used to observe and determine contrast-enhancing of sclerotic lesions.

### 2.3 Segmentation and volumetric analysis

Neuroimaging data were preprocessed using 3DSlicer software, version 5.2.1^1^ (Fedorov et al., 2012), and the extension HD-BET,^2^ a brain extraction algorithm (skull stripping) based on artificial neural networks—this allows a robust analysis even in the presence of neuropathological changes or tissue changes related to patient’s treatment (Isensee et al., 2019). Whole-brain volumetric analysis was performed using a segment statistic tool after brain extraction to quantify the volume of the segment in mm3.

The segmentation of the MS lesions was performed using the 3D Slicer semi-automatic segmentation tool (Fedorov et al., 2012), and the relevant parameter options to adjust the delineation of the margins of the regions of interest were based on T2-FLAIR images.

**FIGURE 1:**
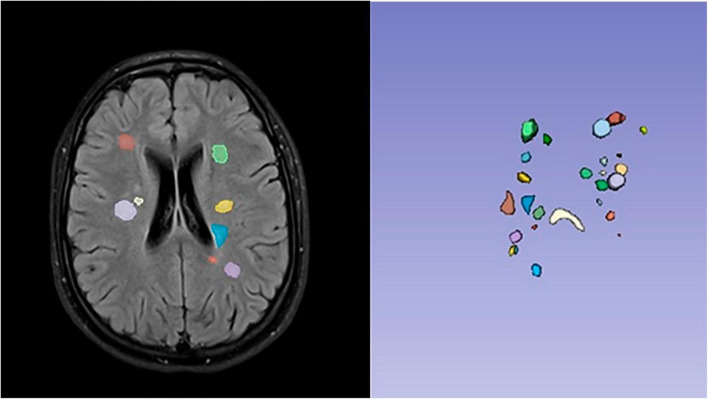
Example patient: segmentation of multiple sclerosis (MS) lesions using 3D Slicer’s semi-automatic tool was guided by T2-FLAIR images. All semi-automated segmentations underwent rigorous validation, including visual inspection by a minimum of three researchers, ensuring accurate and reliable outcomes.

All semi-automatically generated segmentations underwent a thorough validation procedure. Visual inspection, conducted by a minimum of three researchers, was an integral part of this process to ensure the accuracy and reliability of the segmentation outcomes (Figure 1). This meticulous validation approach was particularly feasible given the modest sample size of 11 participants, allowing for comprehensive visual checks on all segmentation results and bolstering the confidence in the correctness of the segmentation procedures.

In addition to lesion segmentation, detailed segmentation of regions surrounding the lesions, extending up to approximately 1 mm, was performed when feasible. The segmentation process, carried out using the 3D Slicer semi-automatic tool and guided by T2-FLAIR images, was executed with precision to avoid including gray matter. Volumes of interest were carefully drawn in the contralateral brain region, enabling the study of unaffected tissue. This comprehensive approach not only facilitated the examination of MS lesions, but also provided valuable insights into the transition zone and contralateral white matter, enhancing the depth of this neuroimaging analysis.

After segmentation, the models module (Fedorov et al., 2012) was used to provide three-dimensional data, which was later used to calculate the volume in mm^3^ of each MS lesion (selecting the “quantification” option and “segment statistics” thereafter). The relationship between brain volume and lesion volume, normalized by lesion size:

$$\mathrm{Difference}\,\mathrm{volume}=\frac{\mathrm{Brain}\,\mathrm{volume}-\mathrm{Total}\,\mathrm{lesion}\,\mathrm{volume}}{\mathrm{Total}\,\mathrm{lesion}\,\mathrm{volume}}$$

, was calculated to determine the difference between volumes.

### 2.4 Radiomics features extraction and analysis

The segmentation used to determine lesions’ volume was used to create the 3D model to extract radiomics features, using the SlicerRadiomics^3^ extension (Pyradiomics library) (van Griethuysen et al., 2017). Pyradiomics is an open-source Python package that was used to calculate a total of 60 radiomics features, organized into the following classes: first-order statistics (19 features), shape-based (17 features), gray-level co-occurrence matrix (GLCM) (24 features) (Lin et al., 2023). In addition to the lesions, regions of interest were also designed to study brain tissue in the transition zone (between the non-lesioned tissue and around each MS lesion), and in contralateral white matter zones without evidence of brain lesions (the results from these ROIs will be referred to as “Normal”).

The methods employed in this study followed the radiomics approach as proposed by Gillies et al. (2016). The analysis of medical images primarily focused on extracting first-order features and features derived from the gray level co-occurrence matrix (GLCM) (Mayerhoefer et al., 2020). For first-order features, we computed fundamental statistics, including mean, median, standard deviation, minimum, and maximum. These parameters provide a comprehensive understanding of the overall distribution of voxel intensities in the studied medical images. Simultaneously, GLCM-based features were utilized to capture more detailed spatial patterns by exploring relationships between the intensity levels of voxel pairs. In this study, the selected radiomic features allowed to highlight texture changes, considering the metrics they provide: “Entropy” represents the randomness in the image values, and it is responsible for measuring the average amount of information needed to encode the values of the image; “SumEntropy” is defined as a sum of the differences in intensity values measured in the neighborhood; “Contrast” is known to be a measure of spatial intensity change; “Autocorrelation” is the metric that represents the magnitude of fineness, allowing the measurement of texture roughness; “ClusterProminence” is known as a measure of skewness and also of asymmetry of the GLCM; “ClusterTendency” feature is responsible for measuring voxel clusters with similar gray level values; and “Variance” is the mean value of the square distances of each intensity value of the mean value. In this study, GLCM was only used to perform the texture analysis, already used in neuroimaging (Dhruv et al., 2019; Korda et al., 2021; Gengeç Benli and Andaç, 2023).

The mean was calculated for all radiomic features extracted from lesions, transitional tissue, and tissue without presence of lesions on T2/FLAIR images. The various characteristics were then subjected to a receiver operating characteristic (ROC) analysis, to identify those with the greatest power to discriminate between the three types of tissue (lesion, transition and normal), considering an area under the curve (AUC) greater than 85% and *p*-value < 0.05. The Youden index was calculated, which simultaneously considers sensitivity and specificity, allowing to find the ideal cut-off point that maximizes discrimination capacity. These surviving characteristics were then used for construction of the classification model through logistic regression (Dieckhaus et al., 2022).

### 2.5 Statistical analysis

Statistical analyses for demographic, clinical, and radiomics data comparison were performed using the Statistical Package for Social Sciences (IBM Corp., Released 2022. IBM SPSS Statistics for Windows, Version 29.0. Armonk, NY: IBM Corp.). Non-parametric tests were used given the small size of the sample, though the ANOVA was retained due to its robustness to small sample sizes and ease of interpretation. The Chi-square (χ2) test was used for comparison of categorical variables, whereas the Mann-Whitney test was used for the comparison of quantitative data. Finally, Spearman’s coefficient was used for studying correlations between demographic, clinical, laboratory values, and radiomic features. The repeated measures ANOVA analysis was conducted to identify significant differences between Lesion, Transition, and Normal tissue groups within subjects. The analysis is useful for highlighting potential statistically significant variations in radiomic patterns, emphasizing the potential clinical relevance of these findings. Statistical significance was considered for *p* < 0.05.

Principal component analysis (PCA) is crucial for dimensionality reduction, providing an in-depth understanding of intrinsic patterns in complex datasets, such as those encountered in radiomic studies. In this research, 60 radiomic features were initially identified, establishing the foundation for subsequent analyses. The implementation of PCA, in conjunction with ROC curve analyses, refined this initial set to a more restricted and informative group of 10 characteristics. This meticulous selection process highlights the most discriminative features, enhancing the interpretation of radiomic data with precision and relevance.

It is important to emphasize that the choice of PCA as an analytical method offers significant advantages. By transforming the original variables into a new set of variables called principal components, PCA provides a more compact and interpretable representation of the data. Each principal component is a weighted linear combination of the original features, representing latent patterns in radiomic characteristics that best explain the variation in the data.

To determine the optimal level of dimensionality reduction while retaining significant information, the cumulative percentage of variance explained by the principal components was considered. It was identified that the first three principal components collectively explained 77.6% of the total variance, with individual contributions of 50.3, 20.5, and 6.9%, respectively. This percentage served as the chosen threshold, as it effectively captured a substantial portion of the dataset’s variability while minimizing information loss.

## 3 Results

### 3.1 Volumetry analysis

The results of the volumetric analysis are shown in Table 2. The volume values (cm3) minimum (Min), maximum (Max), mean, and standard deviation are given for brain volume, total lesion volume, and normalized volume.

**TABLE 2:** Volumetry analysis results.

	Min	Max	Mean	SD
Brain volume	1,216.63	1,709.17	1,480.81	155.90
Total volume Lesion	0.27	10.88	2.84	3.40
Normalized volume	127.01	5,557.79	1,459.95	1,548.48

Volumetric analysis results, showcasing the minimum (Min), maximum (Max), mean, and standard deviation (SD) values for brain volume, total Lesion volume, and Normalized volume.

### 3.2 Tissue group analysis

The results reveal that the analyzed radiomic features provide valuable insights into the differences among lesions, transitional tissues, and normal tissues. These findings (Table 3) highlight the clinical relevance of radiomic features as potential markers to assist in the identification and characterization of pathological tissues, with *p*-values indicating statistical significance and robust eta-squared (η^2^) values (ranging from 0.390 to 0.631), emphasizing the clinical magnitude of the observed variations.

**TABLE 3:** Radiomic features analysis with repeated measures ANOVA.

Radiomic features (GLCM)	Z-value (between groups)	Effect size (η^2^)	Tissue group		*P*-value
Entropy	21.102	0.585	Lesion	Transition	< 0.001
				Normal	< 0.001
Interquartile range	25.644	0.631	Lesion	Transition	< 0.001
				Normal	< 0.001
Mean absolute deviation	22.936	0.605	Lesion	Transition	< 0.001
				Normal	< 0.001
Robust mean absolute deviation	23.595	0.611	Lesion	Transition	< 0.001
				Normal	< 0.001
Sum entropy	24.262	0.618	Lesion	Transition	< 0.001
				Normal	< 0.001
Autocorrelation	11.062	0.424	Lesion	Transition	< 0.001
				Normal	< 0.001
Contrast	9.590	0.390	Lesion	Transition	< 0.001
				Normal	0.006
Cluster prominence	4.812	0.243	Lesion	Transition	0.038
				Normal	0.032
Variance	13.047	0.465	Lesion	Transition	< 0.001
				Normal	< 0.001
Cluster tendency	13.064	0.466	Lesion	Transition	< 0.001
				Normal	< 0.001

Results of a repeated measures analysis of variance (ANOVA) for various radiomic features (GLCM) across different tissue groups, including lesions and transition zones versus normal tissue. The Z-values (between groups), effect sizes (η^2^), and *p*-values are reported for each radiomic feature, indicating significant differences in mean values between the groups. Bonferroni-adjusted multiple comparisons were applied.

### 3.3 Correlations between the volumetry, EDSS score and laboratory data

Table 4 presents Spearman’s correlation coefficients (Rs) for the volumetric data. Brain volume (−0.658, *P* < 0.01) and normalized volume (−0.638, *P* < 0.01) were negatively correlated with EDSS score; total volume lesion (0.547, *P* < 0.01) was positively correlated with EDSS.

**TABLE 4:** Correlations between the volumetry, EDSS score and laboratory data.

	EDSS	CSF oligoclonal bands
Brain volume	−0.658**	0.082
Total volume Lesion	0.547**	−0.577**
Normalized volume	−0.638**	0.577**

Correlations between the volumetry, EDSS score and laboratory data.

**The correlation is significant at 0.01 level.

A significant negative correlation with CSF oligoclonal bands for total lesion volume (−0.577, *P* < 0.01) was also observed, as well as a significant positive correlation for normalized volume (0.577, *P* < 0.01) with the same variable.

### 3.4 Radiomics analysis results

Of the 60 extracted radiomic features, a number of 10 features remained after the principal component and ROC analyses. Data for each of the selected radiomic features are shown in Table 5.

**TABLE 5:** Class and definition of the selected features in neuroimaging data.

	Class	Definition
Entropy	First order	Measures randomness in voxel intensity distribution
InterquartileRange	First order	The range between the first and third quartiles of a dataset, indicating the spread of values.
MeanAbsoluteDeviation	First order	Describes dispersion of voxel intensities
RobustMeanAbsoluteDeviation	First order	A robust version of mean absolute deviation that is less sensitive to outliers.
Variance	First order	A measure of the spread or dispersion of a set of values around their mean.
SumEntropy	GLCM	A measure of randomness in the joint distribution of voxel values in GLCM
Autocorrelation	GLCM	A measure of the similarity between values at different positions in an image.
Contrast	GLCM	GLCM statistic quantifying the difference between the intensities of a voxel and its neighbors.
ClusterProminence	GLCM	Characterizes skewness in distribution of similar intensity voxel pairs
ClusterTendency	GLCM	Measures tendency of voxels to form clusters with similar intensity values.

Class and definition of the selected features in neuroimaging data: class and definition of the selected features extracted from imaging data. All information available on pyradiomics documentation (https://pyradiomics.readthedocs.io/en/latest/).

**FIGURE 2:**
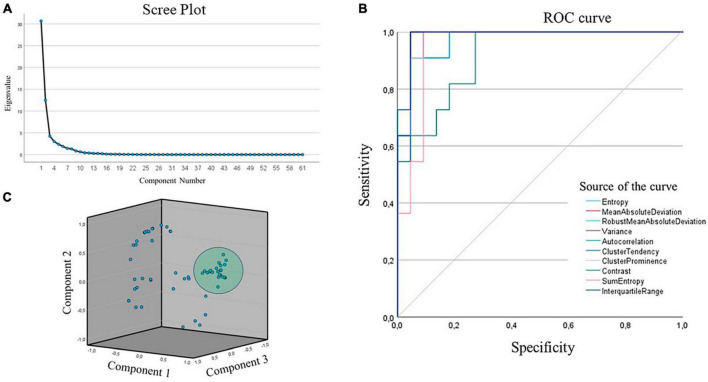
**(A)** Scree plot of the principal components analysis. **(B)** ROC curve after selecting the radiomic characteristics. **(C)** 3D PCA plot of the 3 main components of the analysis.

In the PCA conducted with 8 components, our objective was to identify the most effective radiomic features for discerning lesions from normal tissue (see Figure 2). Notably, we observed that the first three principal components, characterized by eigenvalues of 50.3, 20.5, and 6.9%, collectively explained 77.6% of the variance. These eigenvalues underscore the significance of these three components in representing data, especially in the context of studying radiomic characteristics for lesion identification.

Furthermore, the consistently high values of commonalities for the variables, all exceeding 0.9, signify that a substantial portion of the variance in the original variables, specifically those related to radiomic features, is retained within these main components. This outcome supports the effectiveness of dimensionality reduction in the context of investigating radiomic characteristics.

The principal component analysis outcomes highlight the discernible structure in the data, emphasizing that the first three principal components, with their notable eigenvalues, play a fundamental role in explaining the observed variability, crucial for understanding and identifying lesions relative to normal tissue.

Based on the values of the area under the ROC curve (AUC), the most relevant radiomics features for the classification task were identified. Of the 60 features, 10 features were evidenced with AUCs above 85%, demonstrating the ability to discriminate between classes of interest. The results of the ROC analysis show high sensitivity and specificity in the classification, as evidenced in Table 6.

**TABLE 6:** ROC results.

	AUC	Sensibility	Specificity	YImax	*P*-value
Entropy	0.967	0.909	0.955	0.864	< 0.001
InterquartileRange	0.988	1	0.955	0.955	< 0.001
MeanAbsoluteDeviation	0.983	1	0.955	0.955	< 0.001
RobustMeanAbsoluteDeviation	0.988	1	0.955	0.955	< 0.001
SumEntropy	0.950	1	0.909	0.909	< 0.001
Autocorrelation	0.971	0.909	0.955	0.864	< 0.001
Contrast	0.917	1	0.727	0.727	< 0.001
ClusterProminence	0.975	1	0.909	0.909	< 0.001
Variance	0.983	1	0.955	0.955	< 0.001
ClusterTendency	0.979	1	0.909	0.909	< 0.001

AUC values selected based on the maximum value of the Youden index.

Considering the radiomic features extracted from T2-FLAIR images, a logistic regression was performed to build the classification model and identify the best predictors for MS lesions identification. Nevertheless, the selected variables, namely “InterquartileRange” and “RobustMeanAbsoluteDeviation,” yielded unsatisfactory results in the logistic regression due to extreme odds ratios, indicating a less robust model. For this reason, logistic regressions were conducted for each radiomic variable individually, revealing that the variable “contrast” allows for a statistically significant regression, as indicated by the following model: the obtained logistic model is statistically significant, [χ^2^(1) = 10.278; *p* < 0.001, R^2^Negelkerke = 0.498], (OR = 0.580; *p* < 0.005).

## 4 Discussion results

In this exploratory study, which was conducted using a small sample of POMS patients, as a proof of concept, 10 radiomic features (five first order and five GLCM) were selected from three ROIs: MS lesions, tissue around lesions, and contralateral white matter without the presence of lesions (considered normal brain tissue).

Statistically significant correlations were found between volumetric data and EDSS score. These results agree with a volumetric study performed with twenty-five MS patients, in which conventional spin echo (CSE) and fast fluid attenuated inversion recovery (fFLAIR) sequences were used, that also showed a significant correlation between total lesion volume and EDSS score (Gawne-Cain et al., 1998). Furthermore, Pontillo et al. (2021) showed that predictive models based on features extracted from MRI data have a correlation with clinical disability observed in MS patients.

When considering the possible relationship between total volume of lesions measured by MRI (T2-FLAIR) and the presence of CSF oligoclonal bands, a statistically significant negative correlation was observed; nevertheless, the normalized volume showed a significant positive correlation with that laboratory variable. Recent studies show that patients that test positive for the presence of oligoclonal bands may have a decrease in the volume of the amygdala (Zhao et al., 2020). This study also reports more important microstructural damage in the perilesional normal-appearing white matter (Zhao et al., 2020), which corroborates the interest in investigating, from a radiomic point of view, the perilesional region, which in this study was defined as transitional. In fact, the concept of “smoldering MS” has been debated within the scientific community, which refers to the existence of markers of diffuse inflammatory activity in the brain, far beyond the focal demyelinating lesions that classically characterize the disease (Giovannoni et al., 2022). In this aspect, the perilesional region has been of great interest, as it can, in theory, provide information regarding the activity of the disease and, in this sense, contribute to helping define the timing for an eventual therapeutic adjustment (Giovannoni et al., 2022). In this perspective, it will be interesting, in the future, to design a study focused on deepening knowledge of the radiomics of the perilesional (or transition) region, and their possible correlations with clinical parameters.

As mentioned before, radiomics permits the extraction of large amounts of numerical information from a region of interest defined in medical images (Scapicchio et al., 2021). After a variable selection process, it was found that, in this group of patients, there were 10 parameters that proved to be the most important, 5 first order and 5 belonging to the GLCM class, this one being based on texture, and being used as a biomarker of heterogeneity, from which it is possible to obtain information from the microenvironment of tissues and lesions (Dercle et al., 2017). All of these variables could contribute to increasing knowledge about the biology of MS lesions. The study of its behavior and dynamics, reproduced in imaging exams, has been receiving attention from the scientific community and could constitute another axis of analysis of the pathophysiology of the disease and, necessarily, the way in which available treatments can interfere with it (Moog et al., 2022).

The statistical data derived from radiomic features within regions of interest of the Lesion, Transition, and Normal groups reveal statistically significant differences across multiple metrics. Multiple comparisons suggest consistent variations among groups, indicating distinctions in the radiomic patterns of lesions compared to transitional and normal tissues. For instance, pairwise analyses demonstrate that the mean differences in metrics such as “Entropy,” “Interquartile Range,” “Mean Absolute Deviation,” “Robust Mean Absolute Deviation,” “Sum Entropy,” “Autocorrelation,” “Contrast,” “Cluster Prominence,” “Variance,” and “Cluster Tendency” are all statistically significant, with *p*-values < 0.05. Additionally, the η^2^ values indicate that a substantial proportion of the total variability in these metrics can be attributed to differences between the groups, with η^2^ values ranging from 0.390 to 0.631. These results underscore the clinical relevance of these radiomic features as potential markers to aid in the identification and characterization of pathological tissues.

The characteristics associated with entropy (“SumEntropy,” “Entropy”) showed a statistically significant correlation with the tissue group (lesion, transition and normal), as well as a statistically significant difference between groups (normal-lesion and transition-lesion, *p* < 0.001). These results are in line with other radiomics studies, that show that entropy has an important meaning for the interpretation of tissue heterogeneity, being therefore considered as a potential imaging biomarker, namely in oncology (Dercle et al., 2017), but also in the automatic detection of paramagnetic rim lesions, also seen in adult MS patients (Lou et al., 2021). The possibility of exploring the automation of image analysis, taking into account its radiomic characteristics, is something that could prove to be quite useful in the future, particularly if image characteristics are considered that the human eye is not sensitive to.

The results indicate that the developed model may exhibit a significant performance in classifying multiple sclerosis (MS) lesions, distinguishing them from the other regions of interest (ROIs). However, the actual predictive capacity of the model will need to be investigated in future studies with a different design and a larger sample of participants, considering “Contrast” as one of the predictor variables.

This study has some limitations, namely the small sample size, and it results from the fact that it is only unicentric and exploratory in nature. In radiomics, the predictive classifier model is dependent of a sufficient amount (number) of data. A sample with a minimum of 10 patients has been described as reasonable for a model based on binary classifiers (and that is why data from 11 patients was included, testing the concept that was set out to evaluate) (Gillies et al., 2016). In our future studies, the sample size will be increased, expanding the study to other centers, as well as include more MRI sequences, radiomics functions and perform voxel-based morphometry studies.

## 5 Conclusion

This radiomic approach based on MR imaging (T2-FLAIR) suggest that this model may have a potential contribution to the characterization and classification of demyelinating lesions in POMS, which can facilitate differential diagnosis and eventually contribute to automating some imaging analysis procedures in the future. In addition, it can eventually be a potential tool for understanding the biology of perilesional tissue (whose model also allows classifying), thus contributing to the improvement of the understanding of the pathophysiology of this disease.

## Data availability statement

The original contributions presented in this study are included in this article/supplementary material, further inquiries can be directed to the corresponding author.

## Ethics statement

The studies involving humans were approved by the Local Ethics Committee (Centro Hospitalar e Universitário de Coimbra). The studies were conducted in accordance with the local legislation and institutional requirements. Written informed consent for participation was not required from the participants or the participants’ legal guardians/next of kin in accordance with the national legislation and institutional requirements.

## Author contributions

RF: Conceptualization, Data curation, Formal Analysis, Investigation, Methodology, Project administration, Resources, Software, Supervision, Validation, Visualization, Writing – original draft, Writing – review & editing. CL: Data curation, Software, Writing – review & editing. AJ: Data curation, Software, Writing – review & editing. AM: Data curation, Software, Writing – review & editing. RR: Data curation, Software, Writing – review & editing. CF: Data curation, Software, Writing – review & editing. JF: Data curation, Software, Writing – review & editing. CM: Data curation, Software, Writing – review & editing. AP: Data curation, Software, Writing – review & editing. EA: Data curation, Software, Writing – review & editing. NG: Data curation, Software, Writing – review & editing. LM: Data curation, Software, Writing – review & editing. FP: Conceptualization, Data curation, Investigation, Methodology, Software, Writing – original draft, Writing – review & editing.

## Footnotes

1
https://www.slicer.org/

2
www.neuroai-hd.org

3
https://github.com/aim-harvard/slicerradiomics
